# Parallel locomotor control strategies in mice and flies

**DOI:** 10.1016/j.conb.2022.01.001

**Published:** 2022-02-12

**Authors:** Ana I. Gonçalves, Jacob A. Zavatone-Veth, Megan R. Carey, Damon A. Clark

**Affiliations:** 1 Neuroscience Program, Champalimaud Center for the Unknown, Lisbon, Portugal; 2 Department of Physics, Harvard University, Cambridge, MA, United States; 3 Center for Brain Science, Harvard University, Cambridge, MA, United States; 4 Department of Molecular, Cellular and Developmental Biology, Yale University, New Haven, CT, United States; 5 Department of Physics, Yale University, New Haven, CT, United States; 6 Department of Neuroscience, Yale University, New Haven, CT, United States

## Abstract

Our understanding of the neural basis of locomotor behavior can be informed by careful quantification of animal movement. Classical descriptions of legged locomotion have defined discrete locomotor gaits, characterized by distinct patterns of limb movement. Recent technical advances have enabled increasingly detailed characterization of limb kinematics across many species, imposing tighter constraints on neural control. Here, we highlight striking similarities between coordination patterns observed in two genetic model organisms: the laboratory mouse and *Drosophila*. Both species exhibit continuously-variable coordination patterns with similar low-dimensional structure, suggesting shared principles for limb coordination and descending neural control.

## Introduction

Locomotion is a fundamental animal behavior. It can be initiated or modulated in response to internal needs, such as thirst, hunger, or other internal states, or in response to external stimuli. Although superficially simple, locomotion requires a large number of muscles to work in coordination to create seemingly effortless movement. This coordinated control must be flexible as well as precise, so that an animal can respond to changes in its environment. The space of possible movement sequences is in principle very high-dimensional, but quantifying animal movement can constrain possible neural solutions to this complex control problem [1–6].

**Figure 1:**
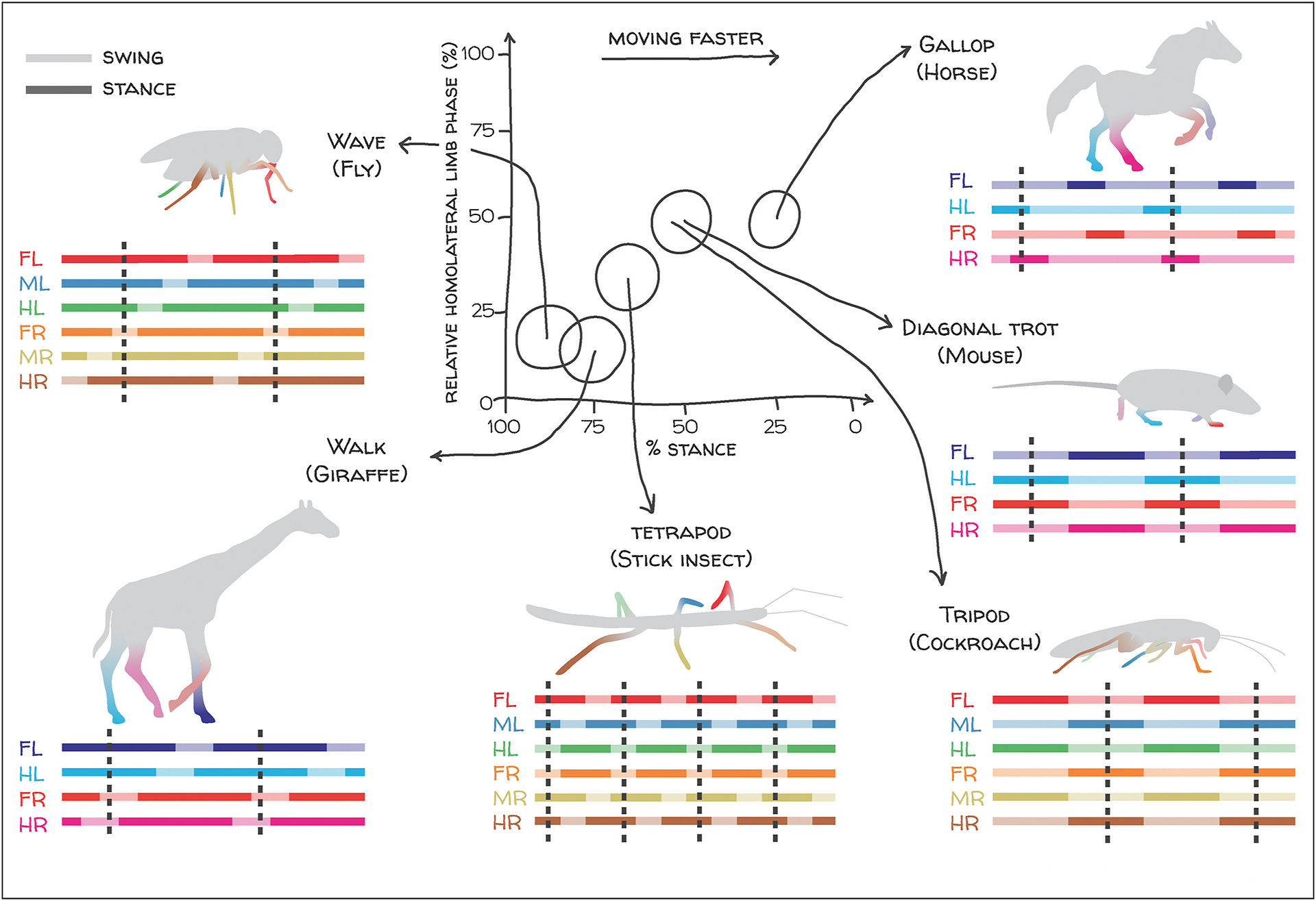
Discrete gaits can be represented by distinct support patterns that depend on the relationship between stance duration and interlimb phasing. In some cases, animals display distinct, preferred patterns of interlimb coordination that can vary depending on size, speed, or species. For example, horses famously alter their gaits at different speeds, with a characteristic gallop at higher speeds. Flies display a wave gait where, at slow speeds, they lift one limb from the ground at a time. Giraffes also have a characteristic slow walk, lifting each limb sequentially. Stick insects display a tetrapod gait, where four limbs touch the ground at each time point in a diagonal arrangement. Cockroaches can show an alternating tripod gait of the six limbs, where diagonal limbs on the ground at the same time. Mice move most of the time in a diagonal trot where one pair of diagonal limbs is in contact with the ground at a time. This figure is modeled after Figure 5 of [17], and uses swing-stance patterns from the studies by Machado et al., DeAngelis et al., and Collins et al. [5,6,10] to estimate the range of relative homolateral limb phases across walking speeds. Canonical stance (solid) and swing (shaded) phases of front-, mid-. and hindlimbs of the left (FL, ML, HL) and right (FR, MR, HR) sides of the body are illustrated for each species.

Historically, variability in locomotor behavior has often been characterized in terms of discrete motifs [4,7–14]. In legged terrestrial animals, modulation of cyclic stepping patterns with forward speed can occur through transitions between distinct gaits, such as trotting and galloping in quadrupeds [7–10,12,15] (Figure 1). Transitions between gaits are characterized by discontinuous changes in limb movement parameters [7,9–11,16–19]. The framework of discrete preferred coordination patterns has strongly influenced models for neural control of locomotion [10,20–24] and analyses of locomotor data [12,18,19,25,26] in animals across many phyla.

Although discrete representations of locomotor patterns can prove useful in summarizing high-dimensional behavior, such representations do not capture the full variability of interlimb coordination patterns. Indeed, even early studies of overground locomotion suggested that patterns may not be truly distinct in all animals, and instead lie along a continuum [7,27]. To accurately constrain neural control mechanisms in different models of animal locomotion, it is therefore critical to fully characterize the variability of their locomotor behaviors.

A complete characterization of locomotor behavior is challenging because the repertoire of these behaviors spans multiple spatial and temporal scales. Modern computer vision techniques can measure the positions of many body parts over time, enabling high-resolution quantification of locomotor kinematics [5,6,26,28–33]. Here, we highlight striking parallels revealed by these measurement advances in the overground locomotor behaviors of a pair of legged animals: the laboratory mouse *Mus musculus* [5] and the vinegar fly *Drosophila melanogaster* [6]. In both species, inter-limb coordination patterns during spontaneous overground locomotion appear largely continuous, without clear evidence for transitions between discrete patterns. The shared low-dimensional structure of coordination patterns suggests that the neural control requirements in these organisms may be similar [34].

## Parallel coordination strategies in flies and mice

**Figure 2:**
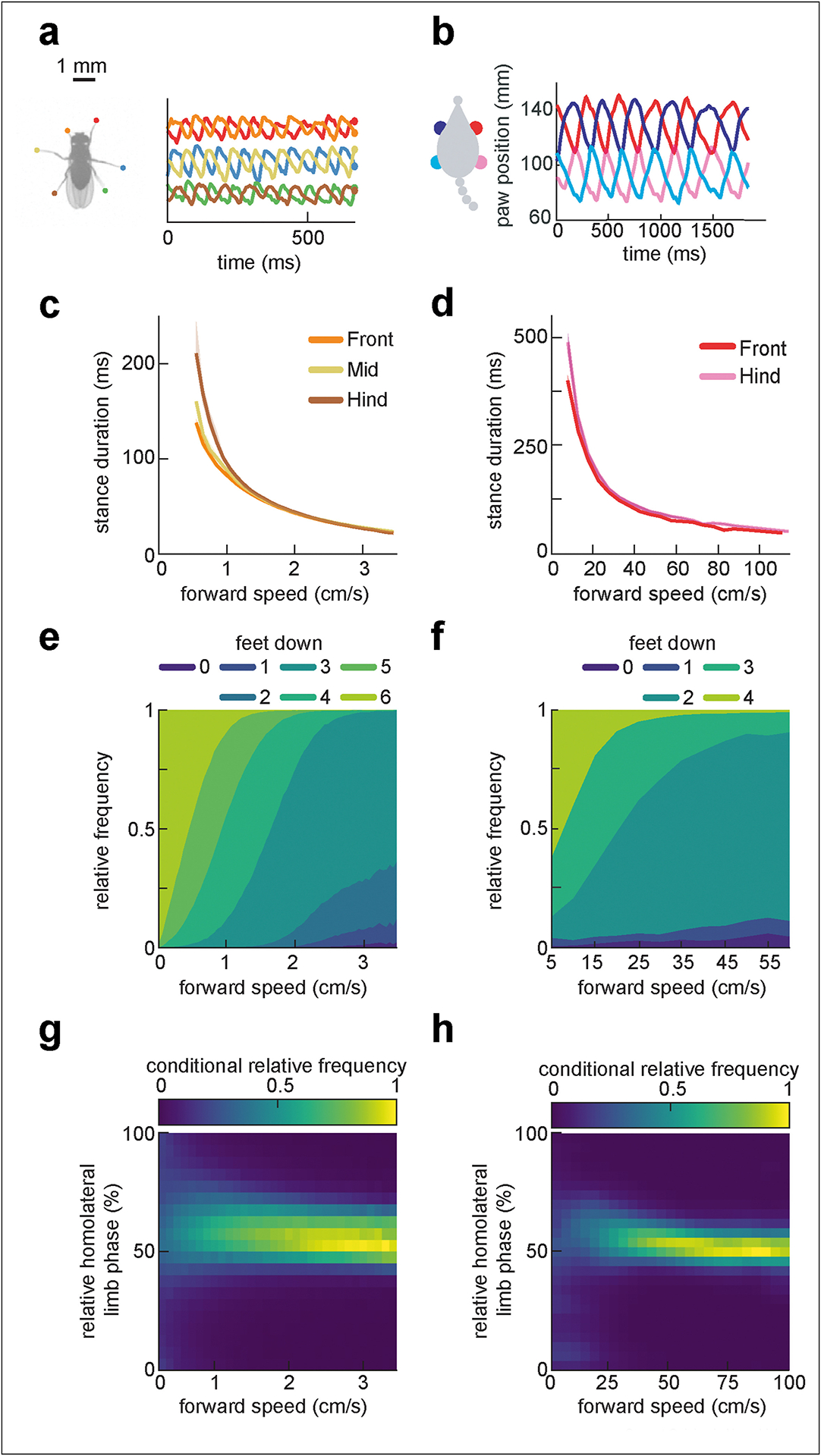
Measurements of fly (left column) and mouse (right column) limb kinematics reveal parallel continua of coordination patterns. (**a, b**) Continuous forward trajectories over time for a fly’s six limb-tips (a) and a mouse’s four limb-tips (aka “paws,” b). (**c, d**) Stance duration decreases steeply with forward walking speed. (**e, f**) Average relative frequencies of limb support patterns within a stride cycle change gradually across forward walking speed for both flies (e) and mice (f; note the expanded speed range). (**g, h**) Speed-conditioned probability distributions of relative homolateral limb phasing vary smoothly and monotonically with forward walking speed for both flies (g, fore-mid claws) and mice (h, front-hind paws). Fly walking data is adapted from the study by DeAngelis et al. [6]; mouse data from the study by Machado et al. [5].

There are in principle many ways in which an animal could modulate its limb kinematics to regulate speed (Figure 1). Despite this possible degeneracy, the two-dimensional locomotor kinematics of mice and flies spontaneously traversing flat terrain are strikingly similar [5,6]. On average, limb-tip (or paw) kinematic parameters in both flies and mice are smoothly modulated as the animal changes its speed (Figure 2). Stride frequency modulation is achieved mostly by altering the duration of the stance phase of the step cycle, when the limb is in contact with the substrate, rather than the swing phase, when it is lifted and extended (Figure 2c–d) [5,6,19,25,26,35–37]. In particular, average stance durations are roughly inversely proportional to forward velocity, while average swing durations vary little and stride length increases roughly linearly with speed. These simple analyses suggest that stance duration may be a dominant dimension of kinematic variability [6,19,38].

Basic metrics of inter-limb coordination also vary smoothly with forward speed. A simple way to characterize inter-limb coordination is by the number of supporting limbs that are in stance phase at a given instant, which remains constant for idealized canonical gaits (Figure 1) [7,10,12]. Consistent with the decrease in stance durations with increasing forward speed, the average instantaneous number of limbs in stance phase decreases with increasing speed in both flies and mice (Figure 2e–f). In both animals, this trend reflects speed-dependent enrichment of certain configurations of supporting limbs in different velocity ranges [5,6,25,26,35–37]. Importantly, the support distributions vary smoothly with speed; there are not sharp transitions between different preferred patterns (Figure 2e–f).

More granular analyses of inter-limb coordination based on relative limb phasing support this common continuum picture. In both flies and mice, distributions of pairwise limb relative phases are unimodal at all forward speeds, with small, smooth monotonic variation in mean relative phases with speed [5,6,37,39] (Figure 2g–h). If multiple preferred distinct coordination patterns were used, one would expect these distributions to be multimodal [10]. Thus, although the interlimb coordination pattern of mice, for example, is slightly more walk-like at slower speeds and more trot-like at faster speeds [5,12], there is no categorical boundary, or distinct gait-switching, associated with increasing speed alone. Therefore, observed distributions of relative phasing do not provide evidence that multiple distinct preferred coordination patterns are used by flies or mice during spontaneous locomotion across a wide range of speeds.

**Figure 3:**
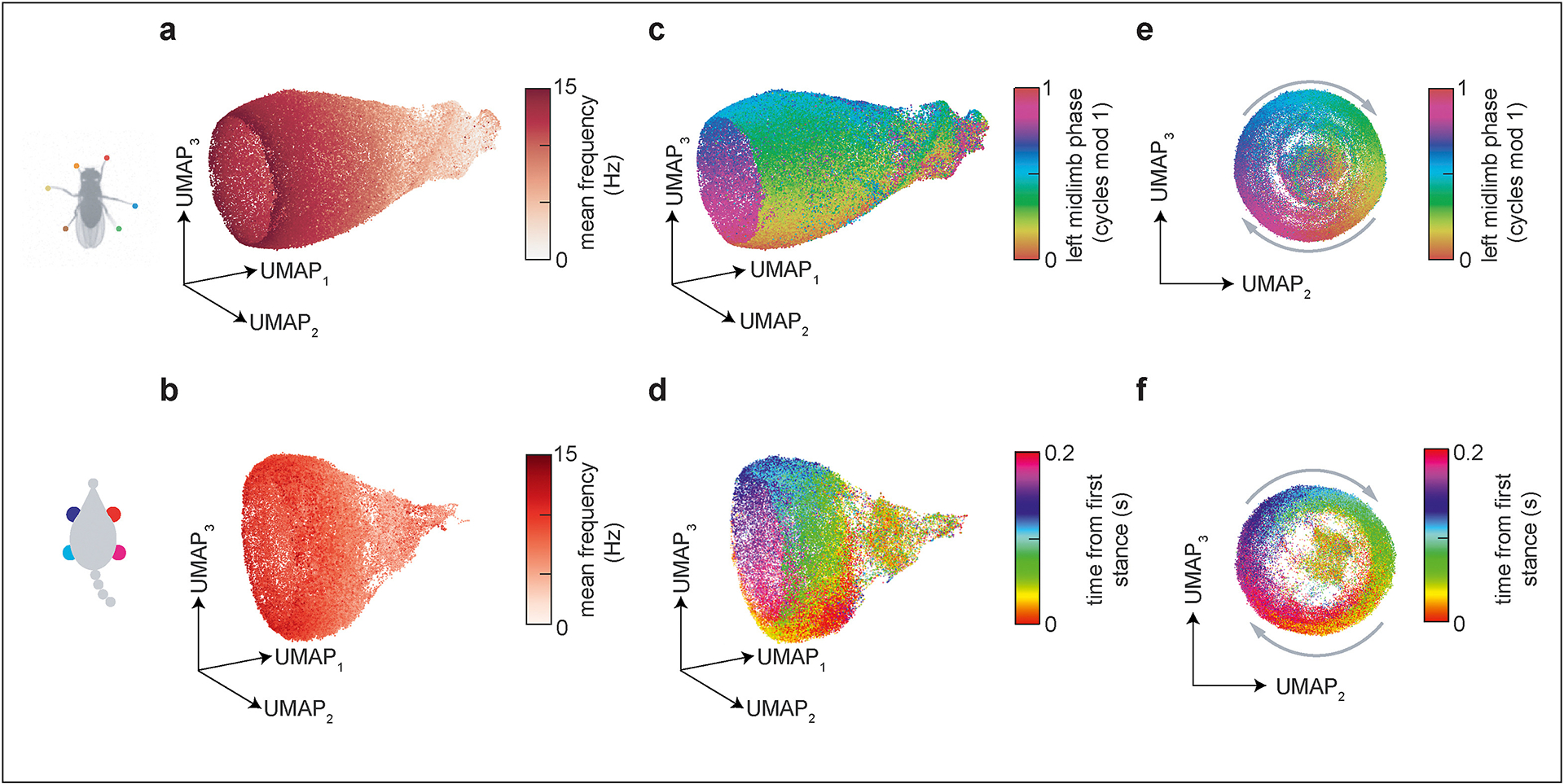
Dimensionality reduction illustrates common low-dimensional structure in fly and mouse interlimb coordination patterns. (**a, b**) Each point in the UMAP embedding represents a random 200 ms segment of limb positions over time, colored by the mean frequency of forward walking for the fly (a) and the mouse (b). (**c**–**f**) UMAP embedding of limb kinematic data colored by instantaneous left mid limb phase for the fly (c). For the mouse (d), colors represent the time of the first stance of the front right paw within each segment as a proxy for limb phase to avoid errors in instantaneous phase estimation due to incomplete information about the stride cycle within individual segments. (e–f) same as (c–d) but illustrating the end-on view of the manifold space. Fly limb coordinate time series embeddings were adapted from the study by DeAngelis et al. [6]. Mouse limb positions over time were collected during tied-belt locomotion on a transparent treadmill, as in the study by Darmohray et al. [40]. As in the study by DeAngelis et al. [6], randomly-sampled segments of limb position timeseries were embedded into three dimensions using a Euclidean distance metric and default UMAP hyperparameters.

Classical metrics of inter-limb coordination show how a subset of features of limb movement change with speed. However, it can be difficult to gain an intuitive understanding for the structure and variability of behavior by manually selecting a small number of features from a high-dimensional dataset. Visualizing such datasets with manifold learning can aid in developing intuition for the structure of behavior [13,14,33]. As in DeAngelis et al. [6], we used the UMAP algorithm [41] to embed segments of fly and mouse limb kinematic timeseries data into three dimensions. In both species, this analysis produced a vase-shaped point cloud, in which the axial dimension corresponds to mean stepping frequency (Figure 3a–b) and the angular dimension corresponds to a global phase (Figure 3c–f). This visualization highlights the similarities between fly and mouse limb coordination strategies, which suggest that they share a common low-dimensional structure.

Here, we have focused on insights into locomotor coordination resulting from kinematic measurements of limb tips (or paws) of animals traversing flat, featureless terrain. More detailed measurements of legged locomotion in more naturalistic environments and in response to external stimuli will further inform neural control mechanisms. Notably, in larger animals, markerless tracking also enables kinematic measurements in the field [29].

## Implications of parallel coordination strategies for descending neural control

**Figure 4:**
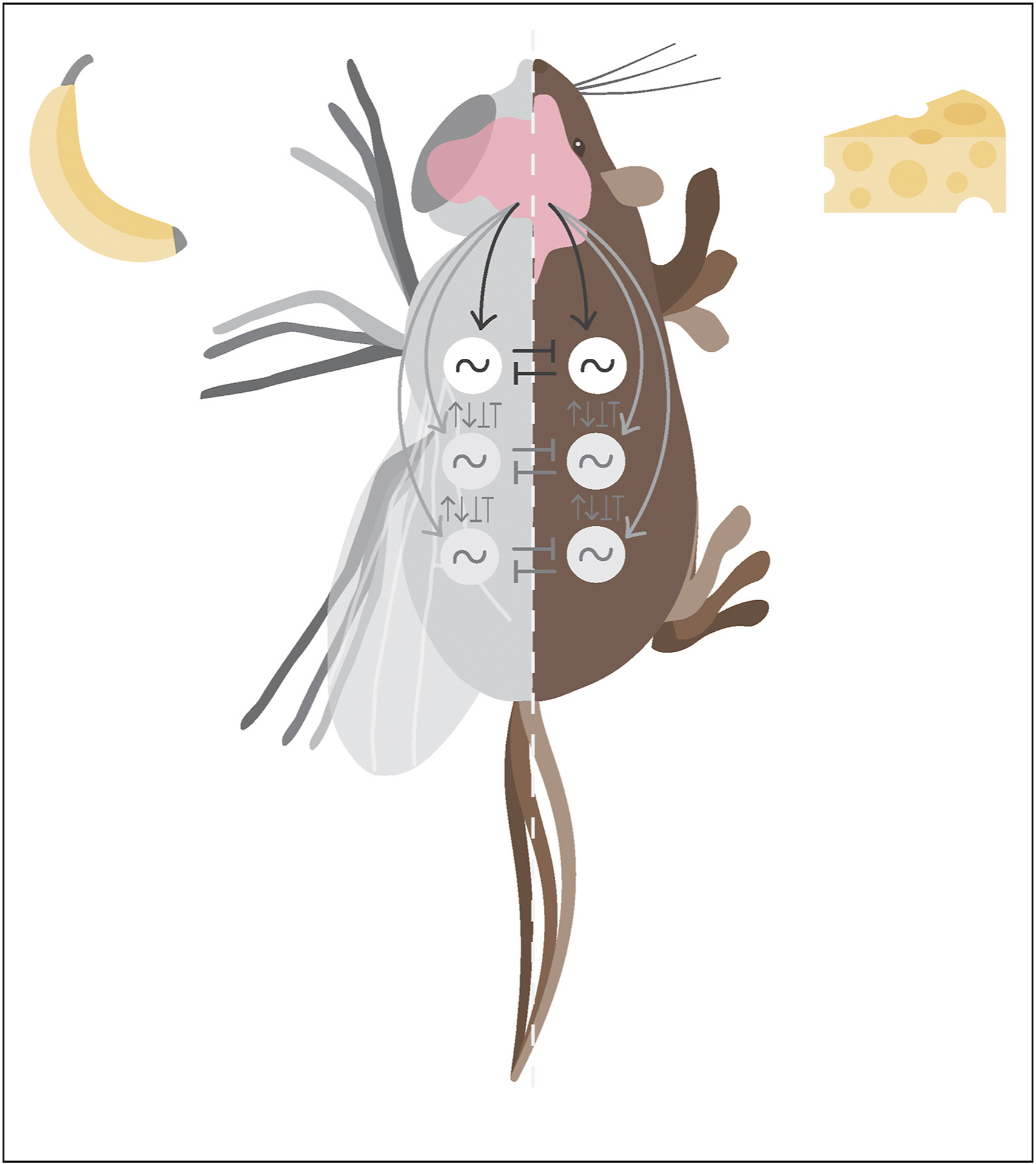
Parallel locomotor control strategies in flies (left) and mice (right). Descending information from the central nervous system, driven by external stimuli, internal state, and/or sensory feedback, modulates locomotor speed and/or direction by modulating CPG modules in the ventral nerve cord of the fly or spinal cord of the mouse, either directly or by modulating internal coupling between CPGs (represented by arrows).

The parallel low-dimensional structures of limb coordination in mice and in flies suggest shared principles for neural control of forward speed modulation [34]. In both species, the oscillatory patterns of neural activity required to produce rhythmic limb movements are believed to be generated by bilaterally-symmetric central pattern generating circuits (CPGs), in the spinal cord of the mouse or the ventral nerve cord of the fly [18,23] (Figure 4). Coordination between limb movements is then established by coupling between CPGs. To smoothly modulate forward speed without causing the animal to deviate from its intended course, these circuits must be modulated symmetrically. The common structure of fly and mouse limb coordination illustrated in Figures 2 and 3 corresponds exactly to this coupled-oscillator idea: the common frequency of the CPGs is modulated continuously, and their oscillation can be summarized by a single global phase [2,10]. Thus, it is possible that one-dimensional command signals could suffice to modulate speed, without the need for detailed descending control of the coupling between CPGs.

Recent studies have begun to dissect descending neural control of forward speed and movement direction in both flies and mice. In mice, supraspinal areas carry instructions to initiate locomotion [42] in a context-dependent setting [43]. Similarly, recent work in flies has identified the set of descending neurons that transmit control signals from the central brain to the ventral nerve cord [44], including individual channels to initiate walking [45–47]. Neurons involved in the termination of locomotion [47–50], speed modulation [6,45,47,49,51], and steering [46,47,52–54] have also been identified in both species. Thus, it seems possible that flies and mice may share parallel principles for descending neural control of locomotion. Similar pathways for controlling the speed [55] and direction [56] of locomotion have also been identified in zebrafish, suggesting that these principles may be more broadly conserved.

In studying how these descending neurons affect changes in limb movements, it will be important to dissect how they modulate the pattern-generating circuitry. In principle, descending commands could directly modulate CPG oscillations [20,21], directly modulate neural couplings between CPGs [22], or indirectly modulate CPGs by altering the gain of sensory feedback. Insects may implement the last of these control strategies [18,57]. In mice there is evidence for all three control strategies. Brain descending inputs send commands to spinal motor circuits modulating locomotor states [43], long-distance projection neurons in the spinal cord couple segments regulating forelimbs and hindlimbs, thus modulating interlimb coordination [58], and sensory feedback is also necessary to modulate locomotor cycle and ongoing movement [59–61].

Another critical supraspinal structure for coordinated movement in vertebrates is the cerebellum. For locomotion, the cerebellum coordinates movements across the body in space and time, sending continuous calibration signals to the spinal cord to ensure that whole-body coordination is maintained and adapted to changes in the environment [5,40,62,63]. In general, the extensive supraspinal control of mouse and vertebrate locomotor circuits likely bestows additional capabilities for behavioral flexibility in diverse contexts and dynamic environments, beyond the scope of continuous forward walking that we have focused on here.

## Outlook

Locomotor behavior is well conserved across legged vertebrate species–for instance, despite large differences in body size, mass and limb configurations, mammals and birds exhibit similar kinematic patterns [64]. In this review, we highlighted the shared low-dimensional, continuous patterns of locomotion in freely walking flies and mice, demonstrating that coordination patterns can be similar across phyla. These findings support the idea that similar kinematic principles and neural control mechanisms may underlie walking in these evolutionarily distant species [34].

Recent developments in tracking technology have the potential to provide a more granular description of locomotor kinematics, including 3D tracking of joint angles across the body [29–32]. More detailed kinematic measurements will provide more stringent constraints on the neural control of locomotion, particularly on the requirements for precise control of individual limbs [31].

Beyond locomotor kinematics, it will be exciting to investigate locomotor dynamics, namely, how locomotor forces contribute to the structure and stability of limb coordination patterns. Though some techniques are available [65–70], measuring and manipulating dynamics remains comparatively challenging. Moreover, these methods have not yet been applied to high-throughput experiments in model organisms. Developing new techniques to measure the dynamics of legged locomotion in naturalistic environments will be an important step towards revealing the interactions between organism and environment that underlie locomotion. High-resolution EMG recording [71] combined with detailed kinematic analysis is likely to be particularly useful here.

Paralleling these opportunities for experimental advances, there exist opportunities for new theoretical work on models of central pattern generating circuits. Thus far, many modeling efforts have focused on incorporating CPGs that support the generation of multiple distinct gaits, and analyzing how low-dimensional descending control signals allow the CPG network to switch between those gaits [10,20–22,24]. As measurements of coordination patterns improve and the underlying neural circuits become better understood, theoretical work will be required to encompass these more complete descriptions of locomotor states. In particular, the observations reviewed here highlight opportunities to develop models that produce continuous sets of inter-limb coordination patterns.

The continuity of inter-limb coordination patterns observed in *Drosophila* and laboratory mouse (Figures 2 and 3) does not imply that all hexapods and quadrupeds share similar control principles. Indeed, many animals exhibit clearly distinct gaits (Figure 1) [4,10–12,15,18]. Moreover, in mice in particular, a broader range of gait patterns can emerge during escape behaviors [12,51], or with genetic perturbations [72]. As detailed behavioral measurements become possible in a broader range of species, it will be important to carefully characterize the full variability of locomotor behavior in each, without importing assumptions from related animals. Careful dissection of behavior, combined with measurement and manipulation of neural activity and with mathematical modeling, is an essential tool for revealing principles for the neural control of locomotor behavior.
